# Perceived Benefits for Mental and Physical Health and Barriers to Horseback Riding Participation. The Analysis among Professional and Amateur Athletes

**DOI:** 10.3390/ijerph17103736

**Published:** 2020-05-25

**Authors:** Ewa Malchrowicz-Mośko, Dariusz Wieliński, Katarzyna Adamczewska

**Affiliations:** 1 Faculty of Sport Sciences, Poznan University of Physical Education, 61-871 Poznań, Poland; wielinski@awf.poznan.pl; 2 Faculty of Health Sciences, Poznan University of Physical Education, 61-871 Poznań, Poland; adamczewska@awf.poznan.pl

**Keywords:** horseback riding, sport participation, sport benefits, sport barriers, sport psychology, sport motivation, sport management, health promotion

## Abstract

The aim of this study was to investigate perceived benefits for mental and physical health and barriers to horseback riding participation among professional and amateur athletes by gender. The empirical study of 2651 professional and amateur horseback riders was conducted during the last edition of Cavaliada competitions (held in Poznan in December 2019)—one of the biggest and most important horseback riding events in Europe. A diagnostic survey method was used in the study. In the questionnaire a division of benefits and barriers according to the EBBS (Exercise Benefits/Barriers Scale) was used. The results are presented by means of frequency distributions for individual items. The verification of hypotheses about the differences between the analyzed groups was conducted using the U-Mann Whitney test with a correction of tied ranks. For the compared groups the mean rank values were calculated. Research results showed that respondents rated the positive impact of equestrianism on mental health higher than on physical health. Among the barriers, the most frequently mentioned aspects were not related to the internal motivation of the respondents, but to external factors—money, time and distance of sports facilities. Men rated the social and psychological benefits higher, while women rated the positive impact of equestrianism on physicality. Professionals rated more highly a number of aspects related to positive effects on the body, while amateurs claimed that were more often not supported by loved ones. This is important research from the point of view of horseback riding promotion. Understanding the horseback riding benefits and barriers are needed, as such knowledge can be used to encourage horseback riding. Perceived benefits and barriers to horseback riding have so far been rarely studied by researchers.

## 1. Introduction

Equestrian sport continues to grow in popularity around the world but previous research on the effects of horseback riding concerns mostly barriers and threats related to injuries associated with this sport [1]. Thompson et al. (2015) presented a multidisciplinary agenda for research that could reduce accident, injury, and death to horse riders around the world [2]. The literature has also centered around the benefits of therapeutic horseback riding for individuals with psychiatric or physical disabilities [3]. Studies on the health benefits for people without these disorders have not yet been conducted. In the era of the growing importance of active recreation for people (including equestrian recreation), it is important to know the perceived benefits to the mental and physical health of contemporary riders, as well as their motivations and barriers for this sport. It is also often claimed that equestrianism is a female-dominated sport [4] and horseback riding tourism provides an opportunity for development of local economies and communities [5,6,7,8]. Thus, it is important to know why people want to undertake this kind of physical activity. Similar research was conducted in other types of sport, e.g., running [9]. Previous studies also centered mainly around motives for participation in mass running events and health effects of this type of physical activity [10,11]. Studies of other sporting disciplines highlight a research gap from this perspective. Some of the studies concerned fan motivations to participate in equestrian competition [12], the welfare of horses [13], and the impact of horse-riding competitions on tourist destinations and local communities [14,15].

The purpose of analyzing the cause-and-effect relationships of women and men who practice horseback riding is to understand the levels at which health is affected, encompassing the human body and mind, for participants. Dhindsa et al. (2008) investigated the impact of an innovative exercise device that simulates horseback riding on cardiovascular and metabolic responses [16]. According to Devienne et al. (2000), regular riding practice and additional physical training are recommended to enhance the physical fitness of competitive riders (dressage and jumping riders) [17]. Bizub et al. (2003) recruited people with mental disabilities for a therapeutic horseback riding program. Beginner riders learned basic horse-riding skills and had the opportunity to bond with an animal. The riders also participated in a post-riding process group that used creative and artistic exercises to promote individual expression. The participants reported success in learning basic horsemanship and also declared additional psychosocial benefits, including an augmented sense of self-efficacy and self-esteem. Researchers claim that this adjunctive therapy can facilitate the recovery process [18]. Yee-Pay et al. (2010) investigated the effectiveness of a simulated developmental horseback riding program using an innovative exercise equipment on the motor proficiency and sensory integrative functions in children with autism. Results showed that children improved motor proficiency and sensory integrative functions [19]. Snider et al. (2007) carried out a systematic review of the literature on horseback riding therapy as an intervention for children with cerebral palsy. They indicated evidence that therapeutic horseback riding is effective for improved gross motor function and that hippotherapy is effective for treating muscle symmetry in the trunk and hip [20]. In addition, Zadnikar and Kastrin (2011) presented an overview of the effects of therapeutic horseback riding and hippotherapy on postural control or balance in children with cerebral palsy. They demonstrated that riding therapy is recommended to improve postural control and balance in children with cerebral palsy [21]. Beinotii et al. (2013) analyzed the impact of horseback riding therapy on the quality of life of people with hemipareses after stroke and they observed significant improvement in the experimental group when compared with the control group [22]. Munoz-Lasa et al. (2011) determined the effect of therapeutic horseback riding on the balance and gait of ambulatory patients with multiple sclerosis. The research results show that therapeutic horse riding can improve balance and gait of ambulatory patients with multiple sclerosis [23]. Therapeutic horseback riding is one form of psychiatric leisure rehabilitation which has been explored for individuals with schizophrenia. Corring and Rudnick (2013) claimed that therapeutic horseback riding benefitted these patients [24]. Kubota et al. (2006) analyzed the acute and chronic effects of exercise on insulin sensitivity in elder diabetic patients using a horseback riding therapeutic equipment. They indicated that in elder diabetic patients, mechanical horseback riding enhances the insulin-induced glucose uptake [25]. The most important beneficial factor of horseback riding for children and for human health appears to be associated with the horse’s vibrations, which may differ among horses. Riding some horses may improve the ability of children to respond with an appropriate action depending on the situation (Go reaction) or use self-control appropriately (No-go reaction), possibly through the activation of the sympathetic nervous system [26].

Worthy of note is the high trauma possibility of this sport. Even for experienced horseback riders, the probability of serious injury while working around horses is 20 times higher than for the motorcyclists. Ridden horses often weigh about 600 kg and can accelerate to about 60 km/h. Such conditions cause a large disproportion between horse and rider. Compared to other sports, horseback riding accounts for the largest number of hospital admittance days by a significant margin. In some countries, 70% of all spine fractures caused by sports activities are related to equestrian activities [27,28]. Nervousness can be transmitted from humans to their horses under riding conditions. It is also vital to know how physical and psychological stress of the humans may influence the horse [29]. As a result of potential risk of injury, horseback riding has been identified as a higher-risk activity than automobile racing, motorcycle riding, football, and skiing, and as being at least as dangerous as rugby [30]. Chest trauma patterns may be a result of significant rider experience [31]. Other studies confirmed that spinal cord injury from horseback riding affects an equal proportion of women and men, has a wide age range, and most commonly results in incomplete tetraplegia followed by complete paraplegia [32]. The use of proper protective equipment and instructions for safe riding should be emphasized [33].

We assumed that barriers and health benefits will differ between equestrian lovers depending on gender. Similar phenomena have been noted in other sports disciplines. For example, based on research conducted among runners, it was found that women have more barriers to spend their leisure time on sport because of high social-family expectations [34]. Tergerson and King indicated that health benefits derived from sport differ by gender. The most commonly reported benefit of exercising among females was to stay in shape, whereas the most commonly reported benefit to exercising among males was to become strong [35]. Our research group consisted of Cavaliada participants. Cavaliada is an international equestrian competition and a large festival, with the goal of promoting horseback riding as a discipline of sport and recreation for all. Cavaliada Tour is a series of international equestrian competitions which take place in Poznań, Lublin, Kraków, and Warszawa, and also is authorized by International Equestrian Federation (FEI). These competitive and show events are highly popular and attract a large number of both competitors and audience participants, numbering in excess of 10,000 people at each event.

The aim of this study is to describe the perceived benefits and barriers to horse riding participation among professional and amateur athletes by gender. It is fundamental to understand the mental, physical, and social benefits and barriers of horse-riding participation to successfully promote this form of physical activity.

## 2. Materials and Methods

### 2.1. Cavaliada Participants

A sample of 2651 horseback riders participated in the study. The respondents were between 18 and 50 years old. Further information about the percentage distribution of the age of respondents are shown in the Table 1. The database was divided into subgroups to ensure that the best possible representation of the results was obtained. The results analysis was conducted in a group of all participants (All, n = 2651) and in the following six subgroups for comparisons:

1. all respondents based on a gender: women (W), (n = 2540/95.8%) vs men (M), (n = 111/4.2%);
2. all respondents based on a specialized degree: amateurs (A), (n = 1906/71.8%) vs professionals (P), (n = 745/28.2%);
3. male respondents based on a specialized degree: amateur men (aM), (n = 79) vs professional men (pM), (n = 32);
4. female respondents based on a specialized degree: amateur women (aW), (n = 1827) vs professional women (pW), (n = 713);
5. amateur group based on a gender: amateur women (Aw), (n = 1827) vs amateur men (Am), (n = 79);
6. professional group based on a gender: professional women (Pw), (n = 713) vs professional men (Pm), (n = 32), *p* < 0.05.

### 2.2. Research Method and Procedure

A diagnostic survey method with a standardized interviewing technique was used (research instrument—online interview questionnaire). The respondents were interviewed during the Cavaliada event. The organizer’s written consent was given to conduct the study. The study was conducted in conformity with the Declaration of Helsinki. As online surveys or questionnaires do not require the completion of a separate participant information sheet or consent form, completion of the survey was deemed to constitute informed consent. Participants were informed about the significance of the study and were kindly requested to provide information. The survey was anonymous, voluntary, and confidential. In Poland, such surveys do not require the consent of the bioethics committee for conducting them among voluntary respondents. A 7-degree Exercise Benefits/Barriers Scale (EBBS) was used in presented study. The instrument has been tested for internal consistency, validity of its constructs, and test-retest reliability [36] and used in many countries, e.g., Brazil and Iran [37,38]. The scale analyzes 14 barriers and 29 benefits in relation to physical activity. The questionnaire had a seven-response, forced choice Likert-type format with responses ranging from 1 (this issue concerns me to a very low degree) to 7 (this issue concerns me to a very high degree). The scale was translated into Polish by authors of the article. The survey was forwarded to Cavaliada participants by the event organizers. The survey was created using Google Docs technology.

### 2.3. Data Analysis

All the statistical analysis was performed using STATISTICA (data analysis software system), version 10 (StatSoft, Inc. 2011, Cracow, Poland). The results are presented by means of frequency distributions for individual categories. The verification of hypotheses about the differences between the analyzed groups was conducted using the U-Mann Whitney test with a correction of tied ranks. In addition, in order to facilitate the interpretation of the presented results, for the compared groups the mean rank values were calculated. Only statistically significant responses were interpreted. The critical level of significance was set at α = 0.05.

## 3. Results

Table 2 presents the percentage distribution of answers given by all respondents (n = 2651) in the range 1–7 (Likert scale).

The highest number of respondents gave the highest rating (7) to the following issues: I enjoy horseback riding (67.9%), My disposition is improved with horse riding (63.6%), Horseback riding makes me feel relaxed (59,68%), Horseback riding improves my mental health (52.92%), and Horseback riding is good entertainment for me (51.04%). Notably, all five top-rated aspects have a psychological dimension and are associated with a positive impact on the mental health of respondents. Among the barriers, the most frequently mentioned aspects at the level of 7 points were the following: It costs too much to go horseback riding (29.27%), Horseback riding takes too much of my time (20.56%), and There are too few places for me to go horseback riding (15.24%). These are not related to the internal motivation of the respondents, but to external factors—money, time and distance of sports facilities.

In the next step we analyzed benefits and barriers divided into three groups with high, moderate, and low rankings (Table 3).

The presented results showed that the respondents declared high rank to 20 aspects (19 benefits and 1 barrier—connected with money), moderate rank to 16 aspects (10 benefits and 6 barriers) and low rank to 7 barriers. It turned out that horse riders gave the highest marks to the benefits of horseback riding and rated most barriers as the least important aspects.

The analysis tried to describe the benefits and barriers differences between sexes. Table 4 shows the motives and barriers of participating in horseback riding based on a gender. Only significant differences in the items between sexes are shown (*p* < 0.05).

Women more often gave higher marks to such benefits as: Horseback riding increases my muscle strength and Horseback riding improves the way my body looks—i.e., aspects related to physicality. However, the following barriers are a bigger problem for them than for men: I am too embarrassed to horseback ride (personal psychological barrier), it is hard work for me (personal physical barrier), My family members do not encourage me to horseback ride, and There are too few places to horseback ride (non-personal aspects connected with lack of family support and insufficient infrastructure).

Men rated more highly aspects such as Horseback riding lets me have contact with friends and Horseback riding is a good way for me to meet new people (social affiliation aspects), Horseback riding helps me to decrease fatigue, and Horseback riding increases my mental alertness. Therefore, men rated the social and psychological benefits higher, while women rated higher the positive impact of equestrianism on physicality.

Table 5 presents the motives and barriers of participating in horseback riding based on specialized degree of the respondents.

Amateurs rated more highly the following aspects: I enjoy horseback riding, Horseback riding improves my health, and My disposition is improved with horse riding. Of greater concern compared to professionals were aspects such as: Places for me to horse ride are too far away, My spouse (or significant other) does not encourage horseback riding, and My family members do not encourage me to horseback ride. Amateurs are therefore more often not supported by loved ones. It can be assumed that in the case of professional sport whole families are involved in horseback riding.

Professionals rated more highly a number of aspects related to positive effects on the body: Horseback riding increases my muscle strength, My muscle tone is improved with horseback riding, Horseback riding improves overall body functioning for me, Horse riding increases my stamina, and My physical endurance is improved by horse riding. Two issues were also rated higher: Horse riding improves my self-concept and Horseback riding increases my mental alertness.

The benefits (potential motives) and barriers of participating in horseback riding based on the specialized degree of male riders are presented in the Table 6.

Male amateurs rated one benefit more highly: Horseback riding decreases feelings of stress and tension for me, and two barriers: My spouse (or significant other) does not encourage me to go horseback riding and My family members do not encourage me to horseback ride.

Male professionals rated more highly the following benefits: Horseback riding gives me a sense of personal accomplishment, Horseback riding increases my level of physical fitness, My muscle tone is improved with horseback riding, My physical endurance is improved by horseback riding, and Horseback riding increases my mental alertness. They also indicated the following problems: Horseback riding is hard work for me, and It costs too much to horse ride.

Therefore, amateur men see the positive impact of this sport on emotions and professional riders have the opportunity to achieve their own goals, but they also have more problems with the high costs of this physically demanding sport.

The differences in benefits and barriers of participating in horseback riding between female respondents based on a specialized degree is shown in Table 7.

Amateur women rated three benefits more highly: I enjoy horseback riding, Horseback riding improves my health, and My disposition is improved with horseback riding; and three barriers: Places for me to horseback ride are too far away, My spouse (or significant other) does not encourage horseback riding, and My family members do not encourage me to horseback ride, while professional women rated five physical aspects more highly: Horseback riding increases my muscle strength, My muscle tone is improved with horseback riding, Horseback riding increases my stamina, My physical endurance is improved by horseback riding, and Horseback riding improves overall body functioning for me.

Table 8 showed the motives and barriers of participating in horseback riding based on specialized degree and gender.

Amateur women rated two benefits more highly: Horseback riding increases my muscle strength and Horseback riding improves the way my body looks, and two barriers: Places for me to horseback ride are too far away and Horseback riding is hard work for me.

Amateur men rated three benefits more highly: Horseback riding lets me have contact with friends and persons I enjoy, Horseback riding is a good way for me to meet new people, and Horseback riding helps me to decrease fatigue.

The benefits and barriers of participating in horseback riding based on specialized degree of gender are presented in the Table 9.

Professional women rated more highly: Places for me to horseback ride are too far away and My family members do not encourage me to horse ride, while professional men rated more highly: Horseback riding is a good way for me to meet new people and Horseback riding increases my mental alertness.

## 4. Discussion

To our knowledge, this is the first comprehensive survey of the motives and barriers to practice horseback riding among professionals and amateurs. Recent studies reported that online studies obtained very similar results as those administered using paper and pencil studies [39,40]. The results of the presented study provide updated information and unique data about this issue. An important conclusion from the research is that all respondents (n = 2651) among the five highest rated benefits mentioned the positive impact of equestrianism on mental health (I enjoy horseback riding, My disposition is improved with horseback riding, Horseback riding makes me feel relaxed, Horseback riding improves my mental health, and Horseback riding is good entertainment for me). Compared to previous studies examining willingness to take part in physical activity, motives and barriers demonstrate both similarities and differences. Some findings suggest that motives for sport participation are more desirable than only those for physical exercise, while, in another studies, general health, and maintaining fitness and good looks, were the most common themes [41,42,43]. Subsequent studies focusing on running emphasize the leading role of emotional and socio-affiliated factors among long-distance runners [44,45]. Our study showed that among the benefits of horse riding, the mental, rather than physical, aspects were rated the highest. Gender-associated differences concerning physical activity are present in the adult population. Caperchione et al. (2013) found that, regarding motivators, men more often reported prevention and reduction of disease and watching others perform. However, reducing weight gain and looking like others were reported more often by women [46]. Taking into account the gender of the respondents in the presented research, men rated the psychological and social benefits higher, while women rated the positive impact of equestrianism on physicality. Interestingly, these results do not coincide with previous studies on other sports disciplines. In the case of cycling, men declared the greater importance of factors related to strength and physical condition, and psychological and social aspects in most cases had similar significance for female and male cycling enthusiasts. In the case of running, women declared a higher importance for emotional factors than men [10,47].

In addition, professionals rated more highly a number of aspects related to the positive effects on the body compared to amateur athletes (and in this group, professional women rated more highly five physical aspects compared to amateur women). Amateur men see the positive impact of this sport on emotions and professional riders have the opportunity to achieve their own goals but they also have greater problems with the high costs of this physically demanding sport. However, among the barriers, amateurs are more often not supported by loved ones. Among the barriers declared by the entire investigated population, the most frequently mentioned aspects at the level of 7 points were the following: It costs too much to go horseback riding, Horseback riding takes too much of my time, and There are too few places for me to go horseback riding. Thus, these factors are not related to the internal motivation of the respondents, but to external factors—money, time, and the distance of sports facilities. Previous studies also point to the significant importance of economic barriers and the problematic distance in the use of sports and recreational infrastructure [48,49].

The key strength of this study is the large sample size, while one limitation involved the use of an online declarative study to obtain the data. In the future, more characteristics of horseback riders should be examined, e.g., age, which is a potentially important factor relating to motivation to go horseback riding. Another issue is the impact of training experience on perceived benefits. In other sport disciplines, the impact of training experience and the way in which a focus on sporting results influences the perception of sport in terms of physical and mental aspects has been examined [50,51]. Such a high assessment of the beneficial effect on mental health of horse riding probably results from the beneficial contact of man with an animal.

The presented study also has some limitations. The main limitation of the research, especially from a statistical perspective, was the number of women and men and amateurs and professionals. Analyzed data presented a large diversity. However, the selected statistical methods allowed for objective comparison of the above analyzed groups.

## 5. Conclusions

This paper refers to psychological and physical benefits and barriers to horseback riding. The majority of sports psychology studies concern other disciplines, e.g., motivation to run, and, regarding equestrianism, most previous studies relate to injuries. An important conclusion from the presented study is that all participants, in the five most-highly rated benefits, mentioned the positive impact of equestrianism on mental health. The influence of equestrianism on the mental sphere is most likely conditioned by the relationship with a live animal, which distinguishes this discipline from others.

There may be important practical implications from the results presented in this study. Not only were the health benefits assessed, but barriers, including the internal mental and physical barriers, were also examined. Taking into account the presented results, the promotion of horseback riding will often be associated with the lifting of barriers that are primarily associated with high costs for participants and fewer places to practice this discipline in large, urban agglomerations. As a result, strategies for overcoming these barriers and to incentivize and assist inactive individuals (especially with urbanization-related mental disorders) to benefit from the presented activity are necessary.

## Figures and Tables

**Table 1:** The percentage age distribution for groups of respondents.

Age			Percentage										
		All	W	M	A	P	aM	pM	aW/Aw	pW	Am	Pw	Pm
	N	2651	2540	111	1906	745	79	32	1827	713	79	713	32
≤25		79.32	80.51	52.25	77.60	83.76	45.57	68.75	78.98	84.43	45.57	84.43	68.75
26–35		11.46	11.26	16.22	11.49	11.41	16.46	15.63	11.28	11.22	16.46	11.22	15.63
36–50		7.95	7.44	19.82	9.44	4.16	22.78	12.50	8.87	3.79	22.78	3.79	12.50
>50		1.24	0.79	11.71	1.47	0.67	15.19	3.13	0.88	0.56	15.19	0.56	3.13

All—all of the participants; W—women; M - men; A —amateurs; P—professionals; aM—amateur men (a group of male respondents based on a specialized degree); pM—professional men (a group of male respondents based on a specialized degree); aW—amateur women (female respondents based on a specialized degree); Aw—amateur women (amateur group based on a gender); pW—professional women (female respondents based on a specialized degree); Am—amateur men (amateur group based on a gender); Pw—professional women (professional group based on a gender); Pm—professional men (professional group based on a gender).

**Table 2:** The percentage distribution of answers given by all respondents (n = 2651) in the range 1–7 Likert scale (1—this issue concerns me to a very low degree; 7—this issue concerns me to a very high degree).

PARTICIPATION IN HORSEBACK RIDING—PERCEIVED BENEFITS (30) AND *BARRIERS (13)*	%						
	1	2	3	4	5	6	7
1. I enjoy horseback riding.	0.04	0.26	0.68	1.70	7.66	21.77	**67.90**
2. Horseback riding decreases feelings of stress and tension for me.	0.53	1.32	3.13	8.53	19.16	25.01	42.32
3. Horseback riding improves my mental health.	0.38	0.64	2.15	6.87	14.11	22.93	**52.92**
*4. Horseback riding takes too much of my time.*	3.73	6.75	10.94	24.63	21.31	12.07	**20.56**
5. I will prevent heart attacks by horseback riding.	24.18	7.54	8.19	25.58	14.90	7.96	11.66
*6. Horseback riding tires me.*	12.45	14.75	17.99	19.50	18.41	10.98	5.92
7. Horseback riding increases my muscle strength.	0.34	1.55	3.85	7.85	20.82	27.84	37.76
8. Horseback riding gives me a sense of personal accomplishment.	5.32	5.85	6.53	10.98	15.84	17.77	37.72
*9. Places for me to horseback riding are too far away.*	25.69	13.92	11.51	14.15	12.60	9.69	12.45
10. Horseback riding makes me feel relaxed.	0.15	0.79	1.21	5.09	12.67	20.41	**59.68**
11. Horseback riding lets me have contact with friends and persons I enjoy.	6.53	6.64	7.47	13.28	16.18	16.71	33.20
*12. I am too embarrassed to horseback riding.*	81.33	8.49	3.21	2.68	2.34	0.87	1.09
13. Horseback riding will keep me from having high blood pressure.	25.05	7.73	10.18	25.46	13.13	7.39	11.05
*14. It costs too much to horseback riding.*	3.09	3.47	6.68	16.75	21.12	19.62	**29.27**
15. Horseback riding increases my level of physical fitness.	0.38	0.87	2.07	6.94	16.90	26.44	46.40
*16. Horseback riding facilities do not have convenient schedules for me.*	57.64	17.01	8.68	8.79	3.89	1.92	2.07
17. My muscle tone is improved with horseback riding.	1.06	1.40	3.62	10.22	19.46	22.86	41.38
18. Horseback riding improves functioning of my cardiovascular system.	8.07	3.96	6.37	19.31	20.56	15.69	26.03
*19. I am fatigued by horseback riding.*	24.48	22.18	18.60	15.62	11.13	5.21	2.79
20. I have improved feelings of well being from horseback riding.	1.47	2.11	3.73	13.35	19.35	20.22	39.76
*21. My spouse (or significant other) does not encourage horseback riding.*	35.50	13.50	9.13	13.62	10.15	7.43	10.68
22. Horseback riding increases my stamina.	0.72	1.13	2.87	8.19	20.18	25.01	41.91
23. Horseback riding improves my flexibility.	2.49	3.47	6.94	14.41	20.29	20.14	32.25
*24. Horseback riding takes too much time from family relationships.*	15.43	13.77	13.84	16.33	14.56	11.88	14.18
25. My disposition is improved with horseback riding.	0.23	0.30	1.24	3.24	10.68	20.71	**63.60**
26. Horseback riding helps me sleep better at night.	7.85	4.00	7.47	16.41	15.73	15.39	33.16
27. I will live longer if I ride.	8.64	4.98	7.28	25.91	16.41	11.39	25.39
*28. I think people in horseback riding clothes look funny.*	51.75	17.13	8.60	8.19	6.56	2.75	5.02
29. Horseback riding helps me decrease fatigue.	9.54	9.66	16.82	25.54	17.31	8.64	12.49
30. Horseback riding is a good way for me to meet new people.	12.41	8.98	9.73	14.98	18.82	13.05	22.03
31. My physical endurance is improved by horseback riding.	1.17	1.51	3.32	10.30	20.33	23.12	40.25
32. Horseback riding improves my self-concept.	6.11	4.49	6.98	17.88	22.03	17.28	25.24
*33. My family members do not encourage me to ride.*	41.19	11.32	7.36	12.45	7.09	6.79	13.81
34. Horseback riding increases my mental alertness.	2.83	3.43	6.87	14.94	21.92	19.84	30.18
35. Horseback riding allows me to carry out normal activities without becoming tired.	5.66	5.81	11.20	20.63	21.61	15.73	19.35
36. Horseback riding improves the quality of my work.	11.35	9.28	13.88	22.26	17.88	10.79	14.56
*37. Horseback riding takes too much time from my family responsibilities.*	23.39	16.07	13.69	15.35	13.54	9.58	8.37
38. Horseback riding is good entertainment for me.	1.17	1.43	2.41	7.81	15.09	21.05	**51.04**
39. Horseback riding increases my acceptance by others.	18.03	12.49	14.82	25.50	13.13	6.87	9.17
40. Horseback riding is hard work for me.	20.75	13.96	14.86	16.94	15.96	9.73	7.81
41. Horseback riding improves overall body functioning for me.	1.77	2.45	4.49	13.58	18.71	21.31	37.68
*42. There are too few places for me to horseback riding.*	16.97	11.05	12.83	18.41	15.13	10.37	**15.24**
43. Horseback riding improves the way my body looks.	5.09	4.34	6.98	15.62	20.33	18.18	29.46

The motives are represented by the normal font; the barriers are represented by italics; the highest rated benefits are represented by bold; the highest rated barriers are underlined. The order of the questions in the table is maintained according to the order of the questions in the original Exercise Benefits/Barriers Scale (EBBS) scale.

**Table 3:** The percentage distribution of answers given by all respondents (n = 2651) in the range 1–7 Likert scale (1—this issue concerns me to a very low degree; 7—this issue concerns me to a very high degree) divided in 3 groups, with low, moderate, and high ranking.

BENEFITS & *BARRIERS* in Descending Order	%		
	1–2 LOW	3–5 MODERATE	6–7 HIGH
HIGH			
1. I enjoy horseback riding.	0.30	10.03	**89.66**
25. My disposition is improved with horseback riding.	0.53	15.16	**84.31**
10. Horseback riding makes me feel relaxed.	0.94	18.97	**80.08**
3. Horseback riding improves my mental health.	1.02	23.12	**75.86**
15. Horseback riding increases my level of physical fitness.	1.24	25.91	**72.84**
38. Horseback riding is good entertainment for me.	2.60	25.31	**72.09**
2. Horseback riding decreases feelings of stress and tension for me.	1.85	30.82	**67.33**
22. Horseback riding increases my stamina.	1.85	31.23	**66.92**
7. Horseback riding increases my muscle strength.	1.89	32.52	**65.60**
17. My muscle tone is improved with horseback riding.	2.45	33.31	**64.24**
31. My physical endurance is improved by horseback riding.	2.68	33.95	**63.37**
20. I have improved feelings of well being from horseback riding.	3.58	36.44	**59.98**
41. Horseback riding improves overall body functioning for me.	4.22	36.78	**59.00**
8. Horseback riding gives me a sense of personal accomplishment.	11.17	33.35	**55.49**
23. Horseback riding improves my flexibility.	5.96	41.64	**52.40**
34. Horseback riding increases my mental alertness.	6.26	43.72	**50.02**
11. Horseback riding lets me have contact with friends and persons I enjoy.	13.16	36.93	**49.91**
*14. It costs too much to horseback riding.*	6.56	44.55	**48.89**
26. Horseback riding helps me sleep better at night.	11.84	39.61	**48.55**
43. Horseback riding improves the way my body looks.	9.43	42.93	**47.64**
MODERATE			
29. Horseback riding helps me decrease fatigue.	19.20	**59.68**	21.12
*4. Horseback riding takes too much of my time.*	10.49	**56.88**	32.63
*6. Horseback riding tires me.*	27.20	**55.90**	16.90
36. Horseback riding improves the quality of my work.	20.63	**54.02**	25.35
35. Horseback riding allows me to carry out normal activities without becoming tired.	11.47	**53.45**	35.08
39. Horseback riding increases my acceptance by others.	30.52	**53.45**	16.03
27. I will live longer if I ride a horse.	13.62	**49.60**	36.78
13. Horseback riding will keep me from having high blood pressure.	32.78	**48.77**	18.45
5. I will prevent heart attacks by horseback riding.	31.72	**48.66**	19.62
*40. Horseback riding is hard work for me.*	34.70	**47.76**	17.54
32. Horseback riding improves my self-concept.	10.60	**46.89**	42.51
*42. There are too few places for me to horseback riding.*	28.03	**46.36**	25.61
18. Horseback riding improves functioning of my cardiovascular system.	12.03	**46.25**	41.72
*24. Horseback riding takes too much time from family relationships.*	29.20	**44.74**	26.07
30. Horseback riding is a good way for me to meet new people.	21.39	**43.53**	35.08
*37. Horseback riding takes too much time from my family responsibilities.*	39.46	**42.59**	17.96
LOW			
*12. I am too embarrassed to horseback riding.*	**89.82**	8.22	1.96
*16. Horseback riding facilities do not have convenient schedules for me.*	**74.65**	21.35	4.00
*28. I think people in horseback riding clothes look funny.*	**68.88**	23.35	7.77
*33. My family members do not encourage me to horseback riding.*	**52.51**	26.90	20.60
*21. My spouse (or significant other) does not encourage horseback riding.*	**49.00**	32.89	18.11
*19. I am fatigued by horseback riding.*	**46.66**	45.34	8.00
*9. Places for me to horseback riding are too far away.*	**39.61**	38.25	22.14

The motives are represented by the normal font; the barriers are represented by italics.

**Table 4:** Comparison of all respondents based on a gender: women (n = 2540) vs men (n = 111), (*p* < 0.05). (1–7 Likert scale: 1—this issue concerns me to a very low degree; 7—this issue concerns me to a very high degree).

BENEFITS & *BARRIERS*	Group	%							Mean Rank	Z	*p*
		1	2	3	4	5	6	7			
7. Horseback riding increases my muscle strength.	W	0.31	1.42	3.82	7.48	20.63	28.43	37.91	1334.60	−2.89	**0.004**
	M	0.90	4.50	4.50	16.22	25.23	14.41	34.23	1129.22		
11. Horseback riding let me have a contact with friends and persons I enjoy.	W	6.69	6.85	7.56	13.27	16.02	16.97	32.64	1316.93	2.99	**0.003**
	M	2.70	1.80	5.41	13.51	19.82	10.81	45.95	1533.53		
12. *I am too embarrassed to horseback ride.*	W	80.98	8.66	3.23	2.68	2.44	0.91	1.10	1330.59	−2.17	**0.030**
	M	89.19	4.50	2.70	2.70	0.00	0.00	0.90	1221.00		
29. Horseback riding helps me to decrease fatigue.	W	9.76	9.45	17.28	25.71	17.17	8.46	12.17	1316.50	3.11	**0.002**
	M	4.50	14.41	6.31	21.62	20.72	12.61	19.82	1543.46		
30. Horseback riding is a good way for me to meet new people.	W	12.48	9.33	9.80	15.08	18.62	13.23	21.46	1316.12	3.22	**0.001**
	M	10.81	0.90	8.11	12.61	23.42	9.01	35.14	1551.99		
33. *My family members do not encourage me to horseback ride.*	W	40.55	11.46	7.40	12.56	7.24	6.85	13.94	1334.49	−2.84	**0.004**
	M	55.86	8.11	6.31	9.91	3.60	5.41	10.81	1131.66		
39. Horseback riding increases my mental alertness.	W	18.11	12.72	14.96	25.63	12.87	6.85	8.86	1317.71	2.71	**0.007**
	M	16.22	7.21	11.71	22.52	18.92	7.21	16.22	1515.64		
40. *Horseback riding is hard work for me.*	W	20.35	13.86	15.12	16.77	16.18	9.84	7.87	1333.17	−2.34	**0.019**
	M	29.73	16.22	9.01	20.72	10.81	7.21	6.31	1161.82		
42. *There are too few places for me to horseback ride.*	W	16.50	11.26	12.83	18.43	15.20	10.31	15.47	1332.19	−2.01	**0.044**
	M	27.93	6.31	12.61	18.02	13.51	11.71	9.91	1184.35		
43. Horseback riding improves the way my body looks.	W	4.88	4.33	6.77	15.39	20.67	18.50	29.45	1332.46	−2.13	**0.034**
	M	9.91	4.50	11.71	20.72	12.61	10.81	29.73	1178.22		

W—women (n = 2540), M—men (n = 111); the motives are represented by normal font; the barriers are represented by italics.

**Table 5:** Comparison of all participants based on a specialized degree: amateurs (n = 1906) vs professionals (n = 745), (*p* < 0.05). (1–7 Likert scale: 1—this issue concerns me to a very low degree; 7—this issue concerns me to a very high degree).

BENEFITS & *BARRIER*	Group	%							Mean Rank	Z	*p*
		1	2	3	4	5	6	7			
1. I enjoy horseback riding.	A	0.00	0.26	0.89	1.47	6.72	20.88	69.78	1352.26	3.44	**0.001**
	P	0.13	0.27	0.13	2.28	10.07	24.03	63.09	1258.82		
3. Horseback riding improves my health.	A	0.47	0.63	2.05	6.30	13.17	22.51	54.88	1354.80	3.39	**0.001**
	P	0.13	0.67	2.42	8.32	16.51	24.03	47.92	1252.33		
7. Horseback riding increases my muscle strength.	A	0.31	1.63	4.04	7.97	21.25	28.96	35.83	1302.24	−2.67	**0.008**
	P	0.40	1.34	3.36	7.52	19.73	24.97	42.68	1386.79		
9. *Places for me to horse ride are too far away.*	A	23.35	14.59	11.80	14.53	12.80	10.28	12.64	1355.01	3.17	**0.002**
	P	31.68	12.21	10.74	13.15	12.08	8.19	11.95	1251.79		
17. My muscle tone is improved with horseback riding.	A	1.10	1.57	3.99	10.65	20.04	22.98	39.66	1297.18	−3.25	**0.001**
	P	0.94	0.94	2.68	9.13	17.99	22.55	45.77	1399.73		
21. *My spouse (or significant other) does not encourage horseback riding.*	A	34.26	13.54	9.29	13.54	10.44	7.29	11.65	1348.62	2.50	**0.012**
	P	38.66	13.42	8.72	13.83	9.40	7.79	8.19	1268.13		
22. Horseback riding increases my stamina.	A	0.79	1.31	3.20	8.55	20.67	25.18	40.29	1298.47	−3.12	**0.002**
	P	0.54	0.67	2.01	7.25	18.93	24.56	46.04	1396.44		
25. My disposition is improved with horseback riding.	A	0.31	0.31	1.36	3.15	9.55	20.46	64.85	1343.74	2.23	**0.026**
	P	0.00	0.27	0.94	3.49	13.56	21.34	60.40	1280.62		
31. My physical endurance is improved by horseback riding	A	1.26	1.73	3.46	10.76	20.62	23.45	38.72	1301.48	−2.76	**0.006**
	P	0.94	0.94	2.95	9.13	19.60	22.28	44.16	1388.73		
33. *My family members do not encourage me to horse ride.*	A	39.45	11.54	7.71	13.17	6.72	6.61	14.80	1350.18	2.71	**0.007**
	P	45.64	10.74	6.44	10.60	8.05	7.25	11.28	1264.13		
36. Horseback riding improves my self- concept.	A	11.44	9.92	14.17	22.30	17.89	10.55	13.75	1306.43	−2.13	**0.033**
	P	11.14	7.65	13.15	22.15	17.85	11.41	16.64	1376.06		
39. Horseback riding increases my mental alertness.	A	18.42	13.27	15.06	24.82	13.12	6.98	8.34	1305.35	−2.26	**0.024**
	P	17.05	10.47	14.23	27.25	13.15	6.58	11.28	1378.83		
41. Horseback riding improves overall body functioning for me.	A	1.73	2.83	4.20	14.38	18.84	21.56	36.46	1307.30	−2.09	**0.037**
	P	1.88	1.48	5.23	11.54	18.39	20.67	40.81	1373.84		

A—amateurs (n = 1906); P—professionals (n = 745); the motives are represented by normal font; the barriers are represented by italics.

**Table 6:** Comparison of male participants based on specialized degree: amateurs (n = 79) vs professionals (n = 32), (*p* < 0.05). (1–7 Likert scale: 1—this issue concerns me to a very low degree; 7—this issue concerns me to a very high degree).

BENEFITS & *BARRIER*	Group	%							Mean Rank	Z	*p*
		1	2	3	4	5	6	7			
2. Horseback riding decreases feelings of stress and tension for me.	aM	0.00	3.80	2.53	5.06	11.39	21.52	55.70	61.93	3.25	**0.001**
	pM	0.00	0.00	15.63	21.88	18.75	15.63	28.13	41.36		
8. Horseback riding gives me a sense of personal accomplishment.	aM	6.33	6.33	11.39	13.92	17.72	10.13	34.18	49.84	−3.31	**0.001**
	pM	0.00	0.00	3.13	6.25	15.63	12.50	62.50	71.20		
14. *It costs too much to horse ride.*	aM	10.13	8.86	12.66	24.05	18.99	10.13	15.19	51.37	−2.42	**0.016**
	pM	12.50	0.00	3.13	18.75	12.50	18.75	34.38	67.44		
15. Horseback riding increases my level of physical fitness.	aM	0.00	1.27	3.80	12.66	20.25	25.32	36.71	51.93	−2.20	**0.027**
	pM	0.00	0.00	0.00	12.50	6.25	21.88	59.38	66.05		
17. My muscle tone is improved with horseback riding.	aM	1.27	3.80	6.33	16.46	18.99	18.99	34.18	51.16	−2.59	**0.010**
	pM	0.00	0.00	3.13	6.25	12.50	21.88	56.25	67.94		
21. *My spouse (or significant other) does not encourage horseback riding.*	aM	39.24	10.13	7.59	12.66	11.39	8.86	10.13	59.72	2.01	**0.045**
	pM	59.38	12.50	3.13	9.38	6.25	0.00	9.38	46.83		
31. My physical endurance is improved by horseback riding	aM	0.00	5.06	3.80	11.39	26.58	20.25	32.91	51.63	−2.35	**0.019**
	pM	0.00	3.13	3.13	3.13	15.63	18.75	56.25	66.80		
33. *My family members do not encourage me to horse ride.*	aM	49.37	8.86	7.59	11.39	5.06	5.06	12.66	59.63	2.05	**0.040**
	pM	71.88	6.25	3.13	6.25	0.00	6.25	6.25	47.05		
39. Horseback riding increases my mental alertness.	aM	16.46	8.86	16.46	20.25	18.99	7.59	11.39	52.23	−1.96	**0.050**
	pM	15.63	3.13	0.00	28.13	18.75	6.25	28.13	65.30		
40. *Horseback riding is hard work for me.*	aM	35.44	17.72	6.33	20.25	10.13	7.59	2.53	51.45	−2.39	**0.017**
	pM	15.63	12.50	15.63	21.88	12.50	6.25	15.63	67.23		

aM—amateur men (n = 79;) pM—professional men (n = 32); the motives are represented by normal font; the barriers are presented by italics.

**Table 7:** Comparison of female participants based on specialized degree: amateurs (n = 1827) vs professionals (n = 713), (*p* < 0.05). (1–7 Likert scale: 1—this issue concerns me to a very low degree; 7—this issue concerns me to a very high degree).

BENEFITS & *BARRIER*	Group	%							Mean Rank	Z	*p*
		1	2	3	4	5	6	7			
1. I enjoy horseback riding.	aW	0.00	0.27	0.93	1.42	6.73	20.96	69.68	1295.02	3.28	**0.001**
	pW	0.14	0.28	0.00	2.24	10.10	24.2	63.11	1207.66		
3. Horseback riding improves my health.	aW	0.44	0.60	2.03	6.24	13.30	22.55	54.84	1296.86	3.17	**0.002**
	pW	0.14	0.70	2.38	7.99	16.41	24.26	48.11	1202.95		
7. Horseback riding increases my muscle strength.	aW	0.27	1.42	4.00	7.66	21.07	29.56	36.02	1248.41	−2.54	**0.011**
	pW	0.42	1.40	3.37	7.01	19.50	25.53	42.78	1327.11		
9. *Places for me to horse ride are too far away.*	aW	22.88	14.67	11.77	14.45	12.81	10.51	12.92	1296.19	2.87	**0.004**
	pW	30.43	12.62	10.80	13.32	12.20	8.27	12.34	1204.68		
17. My muscle tone is improved with horseback riding.	aW	1.09	1.48	3.89	10.40	20.09	23.15	39.90	1246.63	−2.76	**0.006**
	pW	0.98	0.98	2.66	9.26	18.23	22.58	45.30	1331.67		
21. *My spouse (or significant other) does not encourage horseback riding.*	aW	34.04	13.68	9.36	13.57	10.40	7.22	11.71	1289.24	2.12	**0.034**
	pW	37.73	13.46	8.98	14.03	9.54	8.13	8.13	1222.48		
22. Horseback riding increases my stamina.	aW	0.77	1.26	3.12	8.59	20.58	25.12	40.56	1245.37	−2.91	**0.004**
	pW	0.56	0.70	1.96	7.15	18.65	25.25	45.72	1334.90		
25. My disposition is improved with horseback riding.	aW	0.33	0.27	1.42	2.96	9.80	20.14	65.08	1287.59	2.20	**0.028**
	pW	0.00	0.28	0.98	3.23	13.46	21.60	60.45	1226.72		
31. My physical endurance is improved by horseback riding	aW	1.31	1.59	3.45	10.73	20.36	23.59	38.97	1250.40	−2.31	**0.021**
	pW	0.98	0.84	2.95	9.40	19.78	22.44	43.62	1322.00		
33. *My family members do not encourage me to horseback ride.*	aW	39.03	11.66	7.72	13.25	6.79	6.68	14.89	1291.01	2.34	**0.019**
	pW	44.46	10.94	6.59	10.80	8.42	7.29	11.50	1217.94		
41. Horseback riding improves overall body functioning for me.	aW	1.75	2.85	4.16	14.29	18.72	21.84	36.40	1252.24	−2.09	**0.037**
	pW	1.82	1.40	5.05	11.36	18.65	21.18	40.53	1317.30		

aW—amateur women (n = 1827); pW—professional women (n = 713); the motives are represented by normal font; the barriers are represented by italics.

**Table 8:** Comparison of amateur group based on gender: women (n = 1827) vs men (n = 79), (*p* < 0.05). (1–7 Likert scale: 1—this issue concerns me to a very low degree; 7—this issue concerns me to a very high degree).

BENEFITS & *BARRIER*	Group	%							Mean Rank	Z	*p*
		1	2	3	4	5	6	7			
7. Horseback riding increases my muscle strength.	Aw	0.27	1.42	4.00	7.66	21.07	29.56	36.02	960.48	−2.78	**0.006**
	Am	1.27	6.33	5.06	15.19	25.32	15.19	31.65	792.13		
9. *Places for me to horse ride are too far away.*	Aw	22.88	14.67	11.77	14.45	12.81	10.51	12.92	960.23	−2.60	**0.009**
	Am	34.18	12.66	12.66	16.46	12.66	5.06	6.33	797.87		
11. Horseback riding let me have a contact with friends and persons I enjoy.	Aw	7.66	7.61	7.72	13.57	16.75	16.09	30.60	947.52	2.33	**0.020**
	Am	2.53	1.27	7.59	15.19	22.78	7.59	43.04	1091.82		
29. Horseback riding helps me to decrease fatigue.	Aw	7.88	8.54	16.37	26.49	17.95	8.98	13.79	945.87	2.96	**0.003**
	Am	3.80	12.66	6.33	16.46	25.32	12.66	22.78	1129.93		
30. Horseback riding is a good way for me to meet new people.	Aw	13.41	10.40	10.29	15.22	17.95	12.48	20.25	947.28	2.40	**0.016**
	Am	12.66	1.27	7.59	13.92	25.32	7.59	31.65	1097.33		
40. *Horseback riding is hard work for me.*	Aw	23.37	15.27	16.58	16.42	14.89	8.37	5.09	959.17	−2.20	**0.028**
	Am	35.44	17.72	6.33	20.25	10.13	7.59	2.53	822.36		
43. Horseback riding improves the way my body looks.	Aw	4.98	5.04	6.84	16.48	20.63	18.34	27.70	960.49	−2.72	**0.006**
	Am	10.13	3.80	13.92	25.32	13.92	8.86	24.05	791.88		

Aw—amateur women (n = 1827); Am—amateur men (n = 79); the motives are represented by normal font; the barriers are represented by italics.

**Table 9:** Comparison of professional group based on gender: women (n = 713) vs men (n = 32), (*p* < 0.05). (1–7 Likert scale: 1—this issue concerns me to a very low degree; 7—this issue concerns me to a very high degree).

BENEFITS & *BARRIER*	Group	%							Mean Rank	Z	*p*
		1	2	3	4	5	6	7			
9. Places for me to horseback ride are too far away.	Pw	30.43	12.62	10.80	13.32	12.20	8.27	12.34	377.66	−2.85	**0.004**
	Pm	59.38	3.13	9.38	9.38	9.38	6.25	3.13	269.06		
30. Horseback riding is a good way for me to meet new people.	Pw	10.10	6.59	8.56	14.73	20.34	15.15	24.54	369.29	2.26	**0.024**
	Pm	6.25	0.00	9.38	9.38	18.75	12.50	43.75	455.75		
33. *My family members do not encourage me to horse ride.*	Pw	44.46	10.94	6.59	10.80	8.42	7.29	11.50	377.37	−2.76	**0.006**
	Pm	71.88	6.25	3.13	6.25	0.00	6.25	6.25	275.59		
39. Horseback riding increases my mental alertness.	Pw	17.11	10.80	14.87	27.21	12.90	6.59	10.52	368.44	2.78	**0.005**
	Pm	15.63	3.13	0.00	28.13	18.75	6.25	28.13	474.69		

Pw—professional women (n = 713); Pm—professional men (n = 32); the motives are represented by normal font; the barriers are represented by italics.
